# Applying high-performance computing in drug discovery and molecular simulation

**DOI:** 10.1093/nsr/nww003

**Published:** 2016-01-11

**Authors:** Tingting Liu, Dong Lu, Hao Zhang, Mingyue Zheng, Huaiyu Yang, Yechun Xu, Cheng Luo, Weiliang Zhu, Kunqian Yu, Hualiang Jiang

**Affiliations:** State Key Laboratory of Drug Research, Shanghai Institute of Materia Medica, Chinese Academy of Sciences, Shanghai 201203, China

**Keywords:** high-performance computing, computational drug discovery and design, virtual screening, molecular dynamics simulation, protein folding

## Abstract

In recent decades, high-performance computing (HPC) technologies and supercomputers in China have significantly advanced, resulting in remarkable achievements. Computational drug discovery and design, which is based on HPC and combines pharmaceutical chemistry and computational biology, has become a critical approach in drug research and development and is financially supported by the Chinese government. This approach has yielded a series of new algorithms in drug design, as well as new software and databases. This review mainly focuses on the application of HPC to the fields of drug discovery and molecular simulation at the Chinese Academy of Sciences, including virtual drug screening, molecular dynamics simulation, and protein folding. In addition, the potential future application of HPC in precision medicine is briefly discussed.

## INTRODUCTION

According to Moore's law [1], the transistor count of central processing units (CPUs) increases 1-fold every 18–24 months. The performance of supercomputers has developed even more rapidly, and high-performance computing (HPC) has become a strategic technology worldwide [2]. China has made remarkable achievements in the development of supercomputers, under the support of the Chinese government, to which the Tianhe series of supercomputers have recently made significant contributions. Milky Way-1 was designed by the National University of Defense Technology (NUDT) in 1983; its appearance made China the third country to design such supercomputers. In 1994, Dawning-1 was developed by the Institute of Computing Technology of the Chinese Academy of Sciences (CAS), which accelerated the independent development of Chinese supercomputers. In November 2015, Tianhe-2, developed by the NUDT, has retained its position as the world's fastest supercomputer at the International Supercomputing Conference in Austin, America. According to the 46th edition of the twice-yearly TOP500 list of the world's most powerful supercomputers, it has kept the first-place position for six consecutive times since its appearance.

The national HPC environment (NHPCE) has played an important role in the development of HPC applications in China. The Supercomputing Center of the Chinese Academy of Sciences (SCCAS), one of China's earliest established supercomputing centres, is the main node and operating centre of the China National Grid (CNGrid) Service Environment and Supercomputing Environment of CAS (ScGrid). Currently, SCCAS, equipped with the supercomputer ERA with 2.3 PFlops capacity, offers excellent supercomputing services for users in China. Other supercomputing centres, such as in Shanghai, Shenzhen, Tianjin, and Guangzhou, have been supported by 863 national programmes since the 10th Five-Year Plan. These supercomputer centres have more than 1000 users from all over the country, who are not only from basic research fields such as mathematics, physics, chemistry, astronomy, mechanics, biology, medicine, and earth sciences, but are also from important industrial sectors including automotive, aviation, aerospace, nuclear power, steel, shipbuilding, civil engineering, and cartoon rendering. With the improvement of high-performance technologies, HPC and supercomputers are being applied to an increasing number of emerging fields including deep learning, big data mining, computational finance, and precision medicine, with the expectation that it will accelerate innovation.

Computational drug discovery and design (CDDD), also known as computer-assisted drug design, has undergone significant improvements since the 1970s. With the rapid development of molecular and structural biology, the 3D structures and functions of biological macromolecules have been determined. Meanwhile, the application of computational methods to drug discovery and molecular simulation has increased together with the rapid advancement of computer science, powerful functions of graphic workstations, and high-performance computers. Quantum mechanics, molecular mechanics, molecular dynamics (MD), and combinations of these methods have been widely used in drug development. Traditionally, marketing a new drug in the pharmaceutical industry is a difficult process that often costs more than one billion US dollars and takes approximately 10 years. A series of CDDD approaches based on the 3D structures of biological macromolecules (e.g. proteins and nucleic acids), such as the high-throughput virtual screening method, have greatly improved the efficiency of drug discovery. According to a United States government report, because of the application of CDDD, drug development costs are reduced by around 130 million US dollars and research time is shortened by a year [3].

CDDD based on HPC is a combination of pharmaceutical chemistry, computational chemistry, and biology using supercomputers, and has become a critical technology in drug research and development. Many CDDD techniques, such as drug virtual screening, MD simulations, and molecular modelling, can be accelerated by recent technologies including grid computing, cloud computing, and accelerating hardware such as graphics processing units [4]. Grid computing, which is generally composed of several geographically distributed supercomputers, can handle massive computationally intensive tasks [4,5], and is widely used in high-throughput virtual drug screening, thereby reducing time and cost. The drug discovery grid, a project supported by CNGrid, can support massive virtual drug screening tasks [6].

In recent decades, computational drug discovery and molecular simulation in China have significantly advanced. Researchers have developed many new drug design algorithms, as well as drug design software and databases with independent intellectual property, thereby ending a situation of complete dependence on foreign commercial software. Currently, the CDDD field receives consistent financial support from the Chinese government [7]. In addition, more than 90 universities and research institutions all over the country have established departments for computational drug discovery. The Shanghai Institute of Materia Medica (SIMM) at CAS has developed and compiled a series of drug design algorithms and programs, some of which possess thousands of registered users that are currently implanted on supercomputers and are widely used worldwide. For example, the molecular docking software GroupDock, developed by SIMM, has completed parallelization on domestic supercomputers, which has reached hundreds of thousands of CPU cores, allowing virtual screening of large-scale databases in a short time. Besides, the development of GroupDock is based on the UCSF Dock. It mainly deals with the massive docking tasks and optimization of parallelization on China-made HPC systems such as Shenwei. It otherwise performs nearly the same as UCSF DOCK. The software has become a drug research and development platform based on NHPCE, which promotes innovational drug discovery and development. With the help of Chinese supercomputers, SIMM has achieved many things.

**Figure 1.**
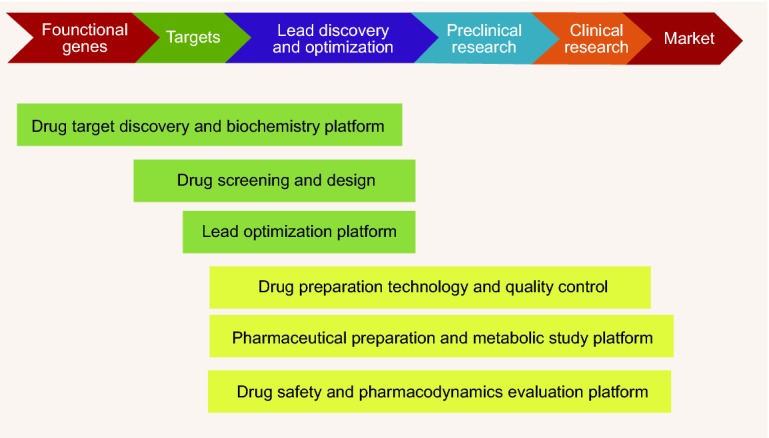
Depiction of the innovative drug research and development system.

SIMM/CAS has established the advanced platforms of innovative drug research and development (Fig. 1). During the implementation of China's 11th and 12th Five-Year Plans, biomedical information and diverse key technology platforms were preliminarily constructed and perfected. These platforms included drug design and screening, preclinical pharmacodynamics of novel drugs, and pharmacokinetics and drug safety evaluations. In addition, computational genomics, computational proteomics, computational regulation networks, and computational drug design are playing an increasing number of important roles in the preclinical stages of the drug development pipeline. Most such computation is computationally intensive and requires the support of HPC supercomputers. By applying HPC, these technology platforms are able to produce biomedical big data that include massive amounts of pharmacological, pharmacokinetic, and pharmacogenomic information with increasing speed. Understanding how to better utilize these data (e.g. huge amounts of public information including patents) and improving our understanding of the complex biological processes underlying drug action and the efficiency of drug discovery are important issues to address for the future of drug development.

Currently, simulation and 3D structure prediction of protein folding, as well as active compound discovery against targets from current compound libraries, have become important directions for drug discovery. The simulation accuracy and enrichment rate of virtual drug screening, to a large extent, depend on computational performance. A supercomputer on the exascale is required for a microsecond simulation of a system containing millions of atoms. Innovative drug-related computational genomics, proteomics, network interaction, and drug design technologies demand mass HPC resources. These critical technologies rely on the development of the next generation of exascale high-performance computers. It is exascale computing that will be in urgent demand for personalized drug development in the next 5–10 years.

This review mainly focuses on the application of HPC to the field of drug discovery and molecular simulation at CAS in recent years, including several cases involving virtual screening (molecular docking), MD simulation, and protein folding. Potential prospects for the application of HPC to precision medicine are briefly discussed.

### Virtual screening and drug discovery

In most cases, the goal of drug discovery is to search for potent inhibitors against a target, using high-throughput screening or virtual screening. The former approach is more reliable, but is expensive and time consuming, whereas the latter is a trade-off between cost and accuracy via the technology of high-performance computers. Thus, high-throughput screening is widely used against well-established targets, mainly in the laboratories of pharmaceutical companies, whereas virtual screening is primarily applied against new or unconfirmed targets, mainly in the laboratories of academic institutions, to discover potent inhibitors and to explore the mechanism of these unconfirmed targets.

#### Drug discovery and validation

**Figure 2.**
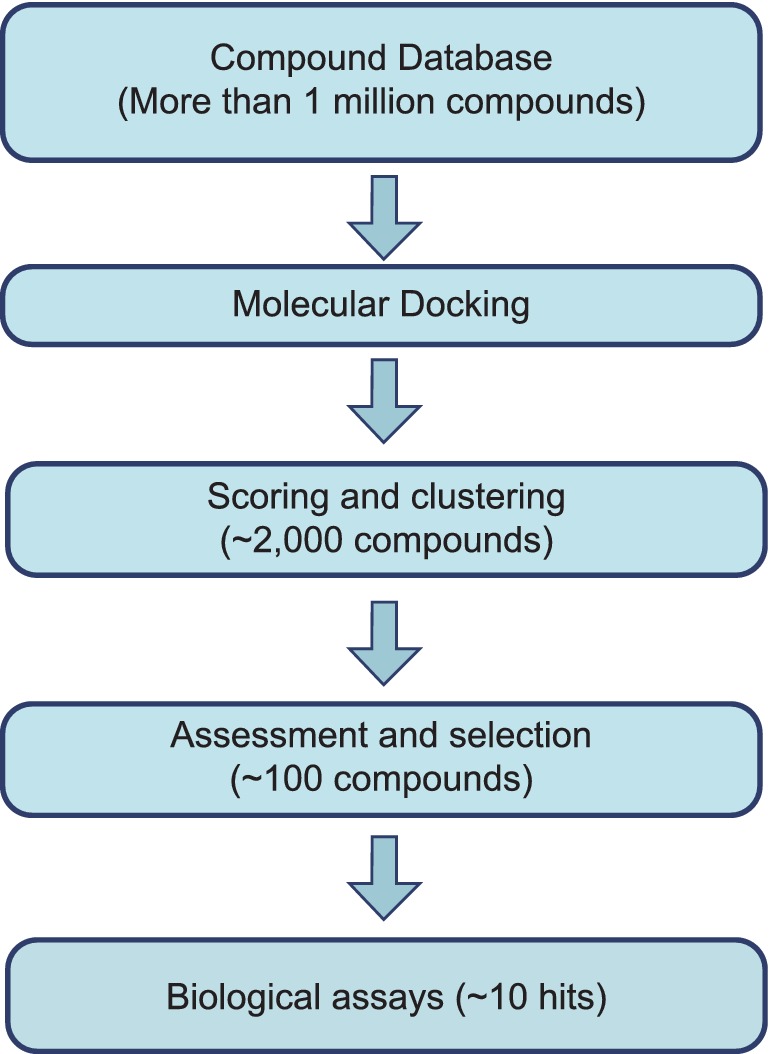
Typical workflow of docking-based virtual screening.

Within a typical workflow of virtual screening, as shown in Fig. 2, a compound database including hundreds of thousands or even millions of small molecule entries is first screened against the designated target protein, via the method of molecular docking. Usually, molecular docking takes a few seconds to minutes on modern CPUs considering only the flexibility of small molecules and keeping the protein rigid. Thus, virtual screening against a target consumes a great amount of computational resources that only supercomputers can provide. Next, thousands of compounds with the best docking scores are kept for structural clustering. Within the clustered compound list, about 100 candidate compounds are finally selected for subsequent validation. The criteria for manual selection are drug-like properties and binding modes generated by molecular docking. To validate the potency of these candidate compounds, various methods are employed, including enzymatic activity assays, enzymatic binding assays, enzymatic selectivity assays, structure–activity relationship studies, and cell assays. Recent successful applications of virtual screening in our lab are briefly introduced below.

Protein arginine methyltransferases (PRMTs) are important for chromatin remodelling, signal transduction, and RNA metabolism. Various diseases, such as cancers and cardiovascular disorders, are associated with the aberrant activity of PRMTs. Through the combination of virtual screening and radioactive methylation assays, several hits were identified as inhibitors with micromolar potency against PRMT1. Among these hits, two compounds showed even higher potency than the well-known PRMT inhibitor AMI-1. They achieved inhibition by directly targeting substrate H4 rather than PRMT1. One of these two compounds also significantly inhibited the proliferation of castrate-resistant prostate cancer cells [8].

The DNA methyltransferases (DNMTs) found in mammals are DNMT1, DNMT3A, and DNMT3B, which are attractive targets in cancer chemotherapy. The first DNMT to be characterized, DNMT1, is responsible for maintaining the DNA methylation pattern of the genome. Most of the existing DNMT inhibitors are nucleoside analogues that can cause toxic side effects. Through a combination of docking-based screening and biochemical assays, a novel non-nucleoside DNMT1 inhibitor named DC_05 was identified, which inhibits DNMT1 at low micromolar concentrations and has remarkable selectivity against other similar methyltransferases. After similarity-based searching, analogues DC_501 and DC_517 showed even higher potency than DC_05. All three compounds significantly inhibited the proliferation of two cancer cell lines [9].

Polycomb repressive complex 2 (PRC2) modulates the structure of chromatin and transcriptional repression by trimethylating lysine 27 of histone H3 (H3K27me3), which is necessary for the protein–protein interaction between the catalytic subunits EZH2 and EED. Aberrant PRC2 activity has been broadly implicated in cancer initiation and progression, making it a promising target for cancer therapy. Through a combination of docking-based virtual screening and biochemical assays, an FDA-approved drug, astemizole, was identified as an inhibitor against the EZH2-EED interaction of PRC2. This work highlighted the therapeutic promise of treating PRC2-driven human cancers via targeted disruption of the EZH2-EED complex [10].

Methicillin-resistant *Staphylococcus aureus* (MRSA) is the principal cause of hospital-acquired infection, which manifests as an infection at the site of surgery, bacteraemia, and sepsis. Frequently, prophylaxis of MRSA infection with antibiotics fails or leads to nosocomial diseases such as *Clostridium difficile* infection, due to drug resistance. Sortase A is a transpeptidase that anchors surface proteins in the envelope of *S. aureus*, and mutant sortases are unable to cause bacteraemia or sepsis in mice. Through virtual screening and structural optimization, a series of inhibitors were identified that blocked the activity of sortase *in vitro* and *in vivo*. These inhibitors protected mice against lethal *S. aureus* bacteraemia without affecting staphylococcal growth *in vitro*. Thus, these sortase inhibitors may act as an anti-infection therapy to protect high-risk patients from hospital-acquired *S. aureus* infection, without incurring the side effects of antibiotics [11].

As a rich source of compounds for drug discovery [12], natural products, especially traditional Chinese medicines (TCM), have been attracting attention in recent years. Various *in silico* methods have been applied in TCM studies to identify potential natural ligands for targets [13]. As an example, the case of molecular docking discovered that sieboldigenin can bind to the pocket of soybean lipoxygenase (SLOX) with an IC_50_ of 38 μM [14]. Artemisia annua (qinghao) was discovered as an inhibitor of SARS-CoVM^pro^ (coronavirus main proteinase) by the combination of molecular docking and fingerprint studies [15]. Besides, several natural product databases based on TCM are available for virtual-screening campaigns, such as Universal Natural Product Database, Chinese Natural Product Database and iSMART [12].

#### Target identification via molecular docking

As the central technology of computer-aided drug design, molecular docking is not only applied in virtual screening for drug discovery, but also aids with target identification. Over the past few years, SIMM has participated in research into target identification with international colleagues using this technology.

**Figure 3.**
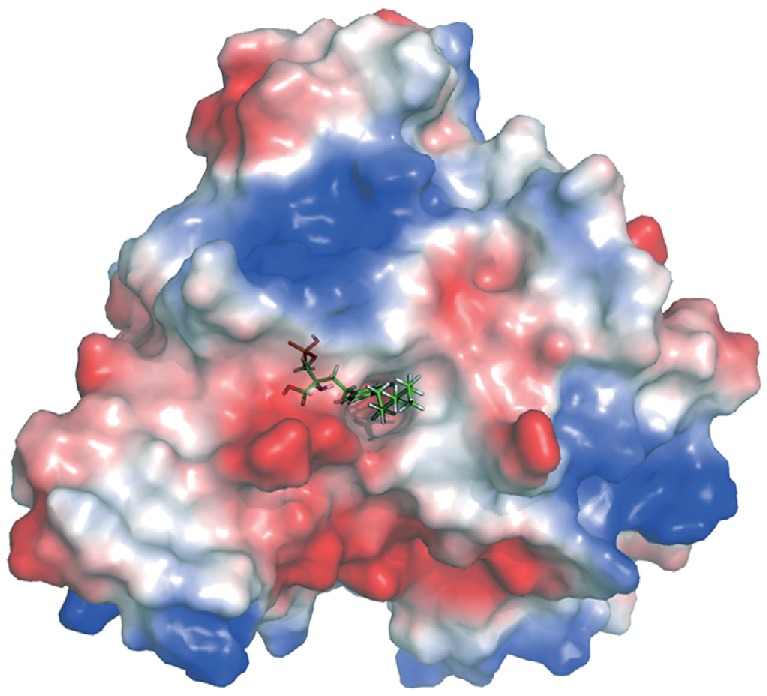
Docking of P-FTY720 into the pocket of HDAC2. The green stick at the centre of figure is P-FTY720. The surface represents HDAC2.

The pleiotropic lipid mediator sphingosine-1-phosphate (S1P) can act independently of its membrane receptors through an intracellular mechanism. Sphingosine kinase 2 (SPHK2), one of the isoenzymes that produce S1P, is associated with histone H3 and regulates histone acetylation via S1P, which specifically binds to the histone deacetylases HDAC1 and HDAC2 to inhibit their enzymatic activity [16]. On the other side, FTY720 (fingolimod), an FDA-approved drug for the treatment of multiple sclerosis, has beneficial effects in the central nervous system that are independent of its effects on immune cell trafficking, although the mechanism is not currently well understood. FTY720 is phosphorylated by SPHK2 in the nucleus, after which it binds and inhibits HDACs, enhancing specific histone acetylation. Having identified the possible binding mode of FTY720 within the pocket of HDAC via molecular docking, as shown in Fig. 3, it was concluded that FTY720 may be a useful adjuvant therapy to facilitate the extinction of aversive memories by targeting HDACs [17].

IRF1 is essential for the IL-1-induced expression of the chemokines CXCL10 and CCL5, which recruit mononuclear cells to the sites of sterile inflammation. Once produced, IRF1 acquires Lys63 (K63)-linked polyubiquitination mediated by the inhibitor of apoptosis cIAP2, which is enhanced by the bioactive lipid S1P. In response to the stimulation of IL-1, cIAP2 and the sphingosine kinase SphK1 (which are responsible for the production of S1P) form a complex with IRF1, leading to its activation. After confirmation that S1P directly binds to cIAP2, molecular docking was used to explore the binding mode of S1P with cIAP2 [18].

Tumour necrosis factor (TNF) receptor-associated factor 2 (TRAF2) is a key component in the NF-κB signalling cascade triggered by TNF-α. TRAF2 binds to Sphk1, which is one of the isoenzymes that produce S1P, a prosurvival mediator in cells. Sphk1 and the production of S1P are necessary for lysine-63-linked polyubiquitination of RIP1 and NF-κB activation. With the molecular docking of S1P into the pocket of TRAF2, this work highlighted the important role of Sphk1 and its product S1P in TNF-α signalling and the canonical NF-κB pathway in inflammatory, anti-apoptotic, and immune processes [19].

#### Target prediction for drug reposition

Drug targets are commonly associated with a desirable therapeutic effect or an unwanted adverse effect [20]. The identification of potential ligand–protein interactions is helpful for predicting therapeutic benefits or the side effects of drug-like molecules. Target prediction and identification is of high significance to drug discovery and research. However, it is a laborious and time-consuming task if undertaken by experimental approaches alone. Thus, there is an urgent need to develop efficient computational methods that can detect the targets of chemical compounds. Recently, Li *et al*. [21] developed a reverse ligand–protein docking programme, TarFisDock, to search for potential ligand–protein interactions through the screening of the potential drug target database. The identification of the enzyme peptide deformylase (the target of natural product *N*-trans-caffeoyltyramine) in *Helicobacter* is regarded as a successful application of TarFisDock [13]. Liu *et al*. proposed a reverse pharmacophore mapping strategy, via which a web server called PharmMapper was established to predict potential drug targets [22].

With the exponential growth of bioactivity data, it has become increasingly difficult to handle the large-scale interaction data of small molecules and their targets via traditional computational strategies. Thus, target prediction can be considered a big data problem. Liu *et al.* [23] developed a ligand-based target fishing method based on 2D fingerprint similarity rankings with data fusion, which is suitable for large-scale drug target prediction because of its simple algorithm and fast calculation speed.

A ligand-target reference library was established that could be updated over time with the increasing availability of bioactivity data. This library currently contains 533 drug targets and 179 807 active ligands, of which each target has its own ligand set. Based on the large reference library, the similarity fusion scores between a query molecule and each ligand set were calculated as the corresponding target scores of this molecule, using the Tanimoto coefficient [24] of ECFP4 fingerprints [25]. Finally, the top targets ranked by fusion scores were considered as the potential targets of the query. A 10-fold cross validation was adopted to evaluate the performance of the similarity fusion schemes or KNN fusion. Similarity Ensemble Approach (SEA) [26], a statistics-based chemoinformatics approach, was also tested in parallel with our fusion schemes for comparison. It was demonstrated that KNN fusion performed better than SEA on a reference set and that 3NN outperformed the most. The target prediction accuracy of the 3NN fusion strategy would be improved with the increasing size of the reference set, which shows that the strategy could be applied to better deal with the problem of big data on active ligands. On the two external test sets compiled from DrugBank and Therapeutic Target Database, the 3NN scheme also exhibited its greater accuracy on the top 20 predictions. Another point of note is that this method could successfully predict 62 of 65 new drug targets identified by SEA.

In order to further verify the effectiveness of the approach on practical cases, target prediction was made for nine drugs withdrawn from the market because of hERG toxicity [27] using the 3NN fusion scheme. In this test, hERG was identified in the top 20 target lists of 7 of 9 drugs, and for terfenadine, sparfloxacin, and droperidol, their interactions with hERG were ranked in first place. Furthermore, the therapeutic targets of nine drugs were all in the top 20. These results demonstrate that the 3NN fusion scheme performs well on the prediction of both therapeutic targets and off-targets.

To make this method more convenient, a user-friendly web server was developed, called TarPred [28], for the prediction of therapeutic benefits and side effects of query molecules. TarPred is freely accessible at http://www.dddc.ac.cn/tarpred. For chemicals queried by users, TarPred accepts both graphical and SMILES format input. After the calculation, which takes only 10–15 minutes, the top 30 interacting targets ranked by 3NN score were listed. TarPred also provides detailed information, such as BindingDB and DrugBank names, Gene IDs and predicted diseases. The disease information associated with targets was extracted from the Comparative Toxicogenomics Database and integrated into the web application. The information provided by TarPred is useful for understanding the functional mechanisms and safety profiles of bioactive ligands. Entecavir and Salvianolic acid B were selected as case studies to validate the effectiveness of TarPred, and the results produced successfully predicted their targets.

As a quick and low-cost method, computer-aided target identification plays an important role in the prediction of new therapeutic targets and potential toxicity for biologically active compounds, which has drawn an increasing amount of attention in recent years. The *in silico* tool TarPred is useful and meaningful for target prediction in several cases. Moreover, it can be applied as a prefilter for drug development and could provide some crucial guidance to drug repurposing. Several online web services for target prediction in China are summarized in Table 1. Several of these web services are supported by HPC servers, which can make the computation time to reduce radically.

**Table 1.** Web servers for target prediction in China.

Server name	Description	Website
TarFisDock	Identification of potential target candidates for a given small molecule via a ligand-protein reverse docking strategy	http://www.dddc.ac.cn/tarfisdock/
PharmMapper	Searches for potential drug targets using the pharmacophore mapping approach	http://59.78.96.61/pharmmapper/
DRAR-CPI [29]	Prediction of adverse drug reactions and rug repositioning based on mining the chemical-protein interactome	http://cpi.bio-x.cn/drar/
iDrug [30]	A versatile web server capable of binding site detection, virtual screening hits identification and drug targets identification	http://lilab.ecust.edu.cn/idrug/
TarPred	Detection of potential ligand-target interactions utilizing 2D fingerprint similarity rankings with data fusion	http://www.dddc.ac.cn/tarpred

In addition, on the platform of the Tianhe-2 supercomputer, SIMM has performed a number of calculations regarding drug repositioning (old drugs for new uses) using large-scale virtual screening.

#### The use of HPC to fight against infectious diseases

Early warning and response to newly emerging infectious diseases before they become pandemic, which is an important part of the national emergency response system, requires the support of HPC. The period of a week to a month after the emergence of a new infectious disease is the most critical time, during which there is usually an absence of effective drugs and vaccines. However, the development of new vaccines takes at least 3 to 6 months, and the traditional process of developing new drugs requires 8–10 years.

Fortunately, drug discovery based on HPC systems can reduce these time periods and boost social confidence in the handling of epidemic situations. For example, in a situation where only small molecule flexibility is considered alongside lower calculation precision, and by employing all the computational resources of Tianhe-2, virtual screening of almost all known compounds on the world can be finished within 1 day. If protein flexibility is considered with higher calculation precision, the current computational power should be elevated by one or two orders of magnitude. That is, exascale calculation can meet the demand.

In the research and development of drugs against major malignant infectious diseases, drug virtual screening becomes more useful due to time limitations and the fact that related experiments cannot be carried out directly. Therefore, upon the sudden outbreak of a new pandemic, the discovery of effective drugs against new diseases from a list of current drugs by applying high-performance computers is an efficacious way of responding to new infectious diseases. We have successfully fought against the SARS [31–36] and influenza viruses [37–44] by employing CDDD approaches and HPC. During the discovery of anti-SARS agents, an *in silico* virtual screening approach was applied to against the small molecules database. As a result, several compounds were discovered as potential drug leads, which offered positive measures against SARS [31,36,45]. CDDD approaches also played an important role in the development of the famous drug Tamiflu, which is now used against the avian influenza viruses.

In the design of drugs against avian influenza viruses, virtual screening was performed by using multiple compound libraries (ACD, CNPD, SPECS) with more than 900 000 compounds, predicting more than 100 potential neuraminidase inhibitors. Computer-aided structural modification and drug synthesis was performed, and nearly 280 analogues of oseltamivir and zanamivir were designed and synthesised. A total of 12 neuraminidase inhibitors of avian influenza were discovered with enzyme activity similar to that of zanamivir and effective *in vivo*. These 12 inhibitors possess independent intellectual property rights, 2 of which have satisfactory activity. Several further lead compounds with innovative structures, independent intellectual property rights, and activities similar to zanamivir (which can act against the virus at a viral level) have been acquired. Furthermore, drug candidates for preclinical studies have been determined. Therefore, it is evident that virtual screening based on HPC systems can remarkably improved the efficiency of drug discovery and drug compound optimisation.

Recently, the virtual screening platform mD3DOCKxb [46] was developed by the combination of NUDT, SIMM, and the National Supercomputer Center in Guangzhou. It is a Many Integrated Core enabled version of D3DOCKxb [46,47]. The authors accelerated the Lamarckian genetic algorithm deeply, and achieved 12× to 18× sppdup. Based on such platform, SIMM carried out a virtual screening project on the Ebola virus on Tianhe-2. Using all of Tianhe-2's resources, a virtual screening task with more than 42 000 000 small chemicals can be completed within 1 day. The combination of HPC and high-efficiency platforms that enabled our country has the ability to deal with the super-high-throughput screening tasks of handling the acute infectious diseases.

### MD simulation

With constant improvements in both computer power and algorithm design, MD simulation has become a powerful research tool for drug discovery over the last few decades. MD simulation calculates the time-dependent behaviour of a molecular system and examines the dynamics of atomic-level phenomena that cannot be observed directly. By exploiting the full power of current HPC platforms on a given scientific problem through effective utilisation of all available resources (CPUs, memory, and in many cases, input/output (I/O)), MD simulation exerts an ever growing role in the life sciences and drug discovery. In the following section, we will discuss our application of MD simulation with the help of three case studies.

#### Identification of dynamic conformations and allosteric binding sites not available from crystal structures

Just as a single photograph of a runner tells little about her stride, the static models produced by NMR and X-ray crystallography provide limited information. Meanwhile, MD simulation can deliver an ensemble of conformations, which promote understanding of the dynamic processes underlying receptor activation [48,49].

**Figure 4.**
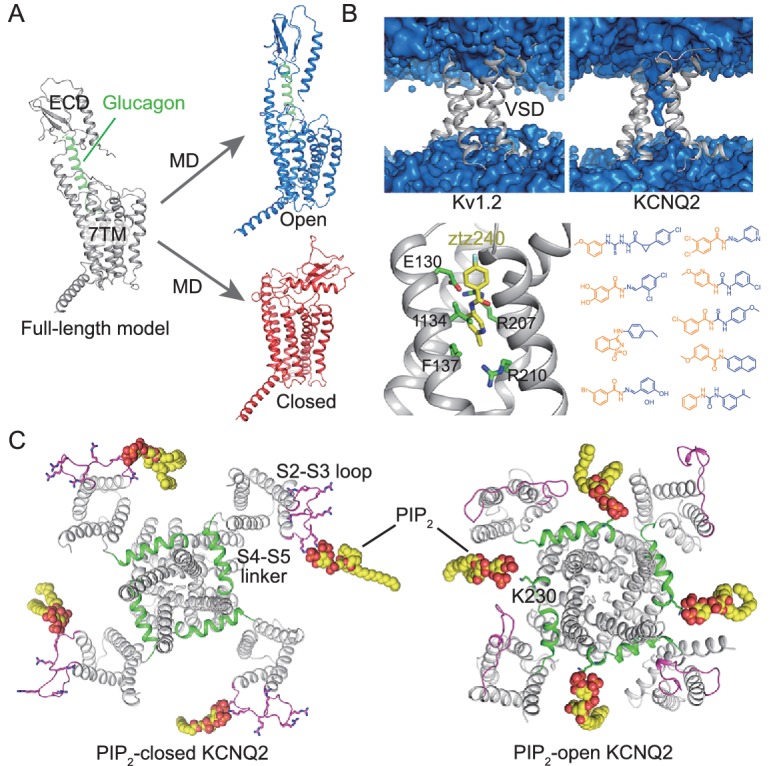
Efficient use of MD simulation applications on HPC infrastructures. (A) Identification of open (blue) and closed (red) states of full-length GCGR in MD simulations of full-length GCGR model (grey). The structures of full-length GCGR and glucagon (green) are shown as cartoons and glucagon is semi-transparent. (B) An activator-binding pocket was discovered in the VSD of the KCNQ2 potassium channel and nine activators were identified through structure-based virtual screening. The surface of water molecules in MD simulations of Kv1.2 and KCNQ2 channels are shown in blue, with VSDs in grey cartoons. Residues involved in interactions with ztz240 in the binding model of ztz240 in the VSD of KCNQ2 are displayed as green sticks. Ztz240 is depicted as yellow sticks. The fragments orientated toward the intracellular end of the VSD in the chemical structures of nine identified activators are highlighted in yellow, while the other ends are highlighted in blue. (C) PIP_2_ interactions with the closed and open states of the KCNQ2 channel in MD simulations. PIP_2_ molecules are depicted as yellow spheres. S2-S3 loops and S4-S5 linkers in KCNQ2 are highlighted in magenta and green, respectively. Residues contributing to the stable binding of PIP_2_ are shown as sticks, such as positively charged residues in the S2-S3 loop and K230 in the S4-S5 linker.

Through 2 μs MD simulations on the apo glucagon receptor (GCGR) and glucagon-bound GCGR performed using GROMACS4.6.1 package [50], we recently discovered that full-length GCGR, a potential drug target for type 2 diabetes [51], has two conformations: an open conformation stabilized by the peptide glucagon and a closed conformation in apo-GCGR, in which the extracellular domain (ECD) forms extensive contacts with the seven transmembrane (7TM) domain (Fig. 4A). The two conformations are very different from the full-length GCGR-glucagon model constructed using crystal structures [52] (Fig. 4A). This was the first report of the putative closed state of GCGR at the atomic level, consistent with hydrogen/deuterium exchange (HDX) results and validated by disulphide cross-linking studies. GCGR belongs to the secretin-like (class B1) family of G-protein-coupled receptors (GPCRs) [53] in humans, which are activated by endogenous hormonal peptides and play causal roles in many diseases, ranging from diabetes and osteoporosis to anxiety. As there are several conserved structural features and ligand interaction hotspots in secretin-like class B GPCRs, the relative movements and interaction dynamics of the ECD and 7TM domain of full-length GCGR provide useful information to guide the design of new experiments to investigate class B GPCR structure–function relationships.

In addition, the protein motions offered by MD simulation provide essential information for the identification of druggable allosteric sites that are not visible in static structures. Previous studies have shown that the voltage-sensor domain (VSD) of Shaker and Kv1.2-2.1 chimaeric channels, which are members of voltage-gated potassium (Kv) channels, is occluded [54–58], as shown in our simulation on the Kv1.2 that there were internal and external water crevices (Fig. 4B). However, in the simulation of the KCNQ2 channel, another member of Kv channels and an anti-epilepsy target, a cavity filled with water molecules appeared in the VSD (Fig. 4B). As the volume of this space is ∼170 Å^3^, we guessed that a potential-binding pocket was located in the KCNQ2 VSD. Based on the docking model of ztz240 (a KCNQ2 activator reported previously [59]) to the KCNQ2 homology model, one MD simulation on the ztz240/KCNQ2 complex was conducted by using GROMACS 4.5.4 package [50], which provided a structural binding model for ztz240. Further mutagenesis and electrophysiological studies validated this activator-binding pocket in the VSD of KCNQ2 [60] (Fig. 4B). Subsequent structure-based virtual screening targeting the identified pocket discovered nine activators with five new chemotypes, two of which exhibited significant anti-epilepsy activity [60] (Fig. 4B). These results demonstrate the capability of the KCNQ2 VSD to accommodate small chemical ligands. The new functionality of the gating charge pathway offers new insights into the therapeutically relevant KCNQ2 channel.

#### Differential regulation of highly homologous receptors by small molecules

Biomacromolecules sharing high-sequence identity, as well as similar structures, play distinct roles in pathology. Beside subtle differences in themselves, the environment they belong to and their interactions with their surroundings, such as small molecules, contribute to their different functions. Because MD simulation enables us to investigate atomic interactions at a very detailed level, it can be successfully applied to identify and predict the differential regulation of highly homologous receptors.

As an example, we will first show its successful application to predict interactions of phosphatidyl-inositol-4,5-bisphosphate (PIP_2_) with the KCNQ2 channel. PIP_2_ is a phospholipid component that is enriched at the plasma membrane, and a substrate for a number of important signalling proteins including the Kv channels [61–64]. Though PIP_2_ alters the physiological function and pharmacological selectivity of KCNQ channels [65–67], the effects and interactions of PIP_2_ on KCNQ channels are not well understood. To predict the interactions of PIP_2_ with the KCNQ2 channel, 200 ns all-atom MD simulations on both the open and closed conformations of the KCNQ2 channel were performed using GROMACS 4.6.1 package [50,68]. Simulations with two different force fields yielded similar PIP_2_ interactions: PIP_2_ preferentially interacted with K230 in the S4–S5 linker of the open-state KCNQ2 channel, whereas it contacted K160 and other positive residues in the S2–S3 loop of the closed state (Fig. 4C). These interactions were different from the PIP_2_–Shaker and PIP_2_–Kv1.2 interactions [69]. Consistently, the effects of PIP_2_ on KCNQ2 were also different relative to the Shaker and Kv1.2 channels. The results indicate the diversity of PIP_2_ effects on Kv channels. The mechanism we proposed for the action of PIP_2_ on the KCNQ2 channel is consistent with the reported subtype sensitivity of PIP_2_ to Kv channels [70,71] and indicates that PIP_2_ may exert specific effects on KCNQ1 channels [68]. Thus, MD simulation is an effective method with which to analyse protein–lipid interaction.

MD simulation has also been successfully applied to identify cholesterol binding for a variety of GPCRs [72–75]. Of interest are the two major β-adrenergic receptor subtypes β1AR and β2AR, which share 76% sequence identity and are highly similar in structure but have distinct and sometimes opposing effects in both cardiovascular physiology and the pathogenesis of heart failure [76,77]. Because β1AR and β2AR are subtypes specifically compartmentalized on cholesterol-enriched caveolae or lipid rafts, two 8 μs MD simulations of β1AR and β2AR embedded in a 2:1 1-palmitoyl-2-oleoyl phosphatidylcholine-cholesterol mixture membrane were performed using the GROMACS 4.5.3 package [50] to compare the potential cholesterol-binding sites for β1AR and β2AR. Big differences were observed in the cholesterol interaction sites between β1AR and β2AR, which are relevant to cholesterol-mediated dimerisation and the allosteric regulation of receptor activity [78]. Our MD simulations provide valuable clues regarding cholesterol binding to β1AR and β2AR and shed light on general determinants of cholesterol binding to GPCRs.

Thanks to the efficient computational resources of the National Supercomputing Center in Jinan, the National Supercomputing Center in Tianjin (Tianhe-1A) and the Tianhe Research and Development Team of the NUDT (Tianhe-2), the abovementioned studies have been accomplished. Because the biological systems studied using all-atom MD simulations can be very large, comprising millions of atoms, the calculations required are often too complex and computationally intensive for even the best supercomputers. It is clear that there is room for improvement. Steady growth in HPC has made MD simulation spans wider spatial and temporal ranges and resolutions, which are likely to play an increasingly important role in the development of novel pharmacological therapeutics.

#### NUMD: a new approach for simulating protein conformational changes and free energy profiles

**Figure 5.**
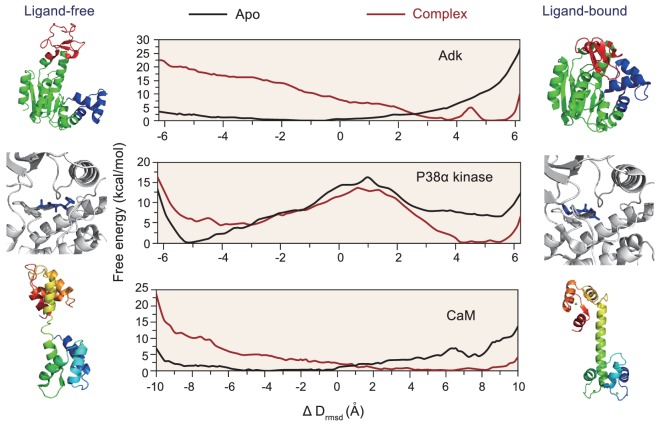
Schematic representations of NUMD application on three well-known protein systems (adenylate kinase (AdK), calmodulin (CaM), and p38 kinase). The ‘apo’ represents the ligand-free conformation, ‘complex’ means ligand-bound state. The order parameterΔD_rmsd_ is defined as the difference in RMSD values of each structure from the reference starting and final states [80].

Large-scale conformational changes of proteins are not only related to the biological functions of the proteins, but are also associated with the binding of ligands or drugs. MD simulation has emerged as a practical approach for exploring protein dynamics but is still unequal as a generally applicable strategy to explore large-scale protein conformational transitions. Considering the structure of the new generation supercomputer, we developed a new approach, called NUMD, to deal with large-scale protein conformational movement, which is a combination of normal mode analysis and umbrella sampling MD simulation. This new approach can produce thousands of the protein conformations along the conformational transition pathway, and each conformation can be simulated simultaneously with thousands of CPUs. Therefore, compared with other widely used MD approaches including NAMD, GROMACS, and AMBER, the new code is specifically suitable for HPC to delineate atomically detailed conformational transition pathways and the associated free energy landscapes. Its application on three well-known protein systems (adenylate kinase (AdK), calmodulin (CaM), and p38α kinase) in the absence and presence of their respective ligands revealed that the predicted conformational transition pathway and thermodynamic observables were in good agreement with experimentally and computationally determined ones (Fig. 5). Thus, this simple approach offers a generally applicable strategy by using HPC for probe intrinsic or ligand-induced large-scale protein conformational transitions and the associated free energy landscape [79,80].

#### Protein folding: conformational transition of amyloid β-peptide (Aβ)

Protein folding is the physical process by which a polypeptide folds into its 3D structure, from a random coil to its characteristic protein functional shape or conformation. The correctly folded protein structure or conformation is essential to the protein's biological function. A failure to fold into the native structure may produce inactive proteins, but misfolded proteins may also have modified or toxic functionality. For example, aggregated proteins are associated with illnesses such as Alzheimer's disease (AD) and Parkinson's disease. MD simulation is a good tool for the study of protein folding and dynamics *in silico*. AD is a progressive, irreversible neurological disorder and the most frequent cause of senile dementia. Two pathologically characterized hallmarks of AD are the appearance of extracellular senile plaques and intracellular neurofibrillary tangles [81,82]. The major constituents of the plaques are amyloid β-peptides (Aβ) of 39–43 amino acids, which are produced by the sequential action of β- and γ-secretases on the amyloid precursor protein [83]. Aβ40 and Aβ42 are the two most abundant Aβ isoforms, with the only difference found at their C-termini, where the latter has two more hydrophobic residues. It has been demonstrated that Aβ42 is more neurotoxic than Aβ40, and that an increased Aβ42/Aβ40 concentration ratio correlates with the onset of AD [84,85].

Extensive experimental and theoretical studies exploring the structures of monomeric as well as aggregated Aβ have revealed remarkable molecular-level insights, including a key point at which the structural feature associated with different morphologies of Aβ are distinct [86–89]. This implies that the peptides are able to adopt a diverse set of conformations in which the anti-parallel or parallel β-sheet is often the key structural element. There is a dire need for structural studies of Aβ monomers and aggregates at the atomic level, to answer crucial questions such as when and how the β-sheet structure is formed and why Aβ42 aggregates more rapidly and severely than Aβ40. In view of the high propensity for self-assembly, MD simulations, complementary to the standard tools of structural biology such as NMR and X-ray diffraction, appear to be a useful tool with which to obtain atomic level insights into the structures as well as the conformational changes of Aβ, a prerequisite to the development of more specific drugs with optimal affinities for Aβ monomers or toxic oligomers.

**Figure 6.**
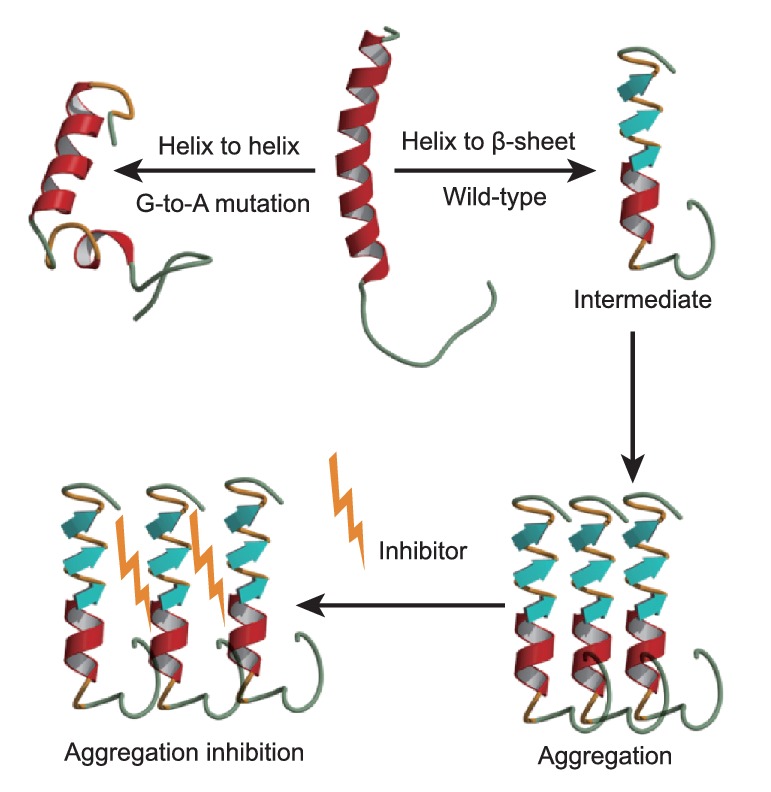
MD simulations of the conformational transition of Aβ and design of an aggregation inhibitor targeting the intermediate structure of Aβ resulting from simulations.

We performed multiple parallel MD simulations with a total of 850 ns on both the wild-type peptide and its mutant [90]. In aqueous solution, an α-helix to β-sheet conformational transition of Aβ40 was observed and a completed unfolding process of the peptide from helix to coil was traced by our MD simulations. Structures with few short β-sheets and helices were identified as intermediate states in the unfolding pathway of Aβ40 (Fig. 6). Four tandem glycines (G25, G29, G33, and G37) at the C-terminal of Aβ40 were found to account for the short β-sheet formation. Further mutation of these glycines to alanines almost abolished the β-sheet formation and increased the content of the helix component instead (Fig. 6). In the lipid bilayer of dipalmitoyl phosphatidylcholine, the major secondary structure of Aβ40 is helical; however, the peptide tends to exit from the hydrophobic region and lie down on the surface of the bilayer. Overall, these results provide an annotation of conformational transition in relation to the dynamic process of Aβ exiting from the membrane and entering into aqueous solution. It is beneficial to understand the underlying molecular mechanism of Aβ aggregation and design compounds that can inhibit the formation of oligomers as well as amyloid fibrils (Fig. 6).

The α-helix/β-sheet intermediate structure revealed by our simulations has a core domain constituted by the segment of residues 24–37, of which four residues (Gly25, Gly29, Gly33, and Gly37) are essential to β-sheet formation. Chemical compounds that lock the structure of Aβ into the α-helix/β-sheet intermediate state could possibly inhibit Aβ fibrillation. Targeting this intermediate structure, a virtual screening based on molecular docking was performed with the goal of discovering small molecule inhibitors that interrupt the formation of the pleated β-sheet structures involved in amyloid fibrils via binding to the intermediate structure [91]. One of the docked compounds, DC-AB1, was shown to effectively inhibit the aggregation and fibrillation of Aβ by thioflavin T fluorescence assay, atomic force microscopy, and polyacrylamide gel electrophoresis analysis. Meanwhile, CD spectroscopy measurement revealed that the β-sheet component of Aβ increased in the presence of DC-AB1, suggesting that the binding of DC-AB1 to Aβ stabilized the β-sheet structure of the peptide. Taken together, our study not only demonstrated that the α-helix/β-sheet intermediate structures revealed by the aforementioned simulations could be used as a binding target for inhibitor design, but also provided new insights into the molecular events involved in the conformational transition of Aβ peptides in fibrillogenesis.

Subsequent structure optimization of DC-AB1 was performed and nine compounds with higher potency were obtained [92]. In particular, one of the nine new compounds not only suppressed the aggregation of Aβ but also dissolved the preformed fibrils *in vitro*. Cellular assays revealed that it has no toxicity to neuronal cells. Moreover, it can effectively inhibit Aβ42-induced neurotoxicity and increase cell viability. Therefore, based upon the intermediate structure of Aβ revealed by MD simulations, virtual screening together with further structure optimization has resulted in a lead compound for the development of a drug against AD.

Characterizing the conformation dynamics of monomeric Aβ40 and Aβ42 is a prerequisite to comprehending the Aβ self-assembly pathway and the differences between the two peptides. In a recent study, we set out to investigate the conformation dynamics of Aβ40 and Aβ42 by exploring the impact of intramolecular interactions on conformation dynamics using equilibrium MD simulations [93]. Our 40 μs-scale simulations revealed heterogeneous conformation ensembles of Aβ40 and Aβ42 that encompassed ∼35% β-strand and ∼60% unstructured coils. Two conformational states were identified in both isoforms: a collapsed state (CS) that resembles the structural motif of face-to-face hydrophobic clustering in amyloid fibrils, and an extended state (ES) that carries the structural feature of anti-parallel β-sheets observed in amyloid oligomers. This observation is in line with previous NMR studies. In Aβ40, the C-terminus remains unstructured and rarely interacts with other parts, therefore, the hydrophobic clustering is loosely structured and the peptide assumes an ES with high probability. In contrast, the C-terminus of Aβ42 adopts a β-strand structure that strongly interacts with segments E3-R5 and V18-A21. The active associations of this extra β-strand lead to a more compact hydrophobic collapse and prevent the isoform from forming the ES. Based on our structural characterization, we propose that the aggregation begins in the CS, and that the propensity of the various isoforms to self-associate into a particular form is encoded in their equilibrium conformational states. As our simulation results demonstrate that the probability of Aβ42 staying in CS is significantly higher than that of Aβ40, the enhanced aggregation propensity of the former could result from the consequent lowered energy barrier for nucleate formation. Besides, a greater resemblance to fibril structure is observed in the CS of Aβ42, including increased occupancy of the β-strand at V18-A21 and I31-M35 and compact hydrophobic collapse. These structural advantages could also contribute to the effectiveness of nucleation.

### Future perspectives: personalized drug discovery (precision medicine) based on HPC

Precision medicine is also known as ‘P4 medicine’ [94], which refers to predictive, personalized, preventive, and participatory medicine. Owing to the rapid pace of human genome research and high-throughput technologies, personalized medicine is expected to become a new paradigm of future healthcare [95]. Personalized medicine will be likely to affect society in many aspects [96]. Radical changes to medical practice and the pharmaceutical industry will be involved.

Currently, personalized medicine is leading the third wave of drug discovery. The United States introduced a ‘precision medicine initiative’ in early 2015, in order to promote the development of personalized medicine, hoping to lead a new era of medication and healthcare. The rapid development of supercomputers will play a significant role in the development of ‘precision medicine’, and will greatly strengthen the computation and data analysis ability of genomics. Under the vigorous promotion of high-performance computers, precision medicine based on genomics and personalized drug research and discovery should greatly improve therapeutic effects, by employing personalized accurate treatment of ‘the remedy to the case’. The discovery and validation of functional genes and targets in the early stages of drug research and development lead discovery and optimization, and the platform of drug virtual screening and design must be based on HPC infrastructure.

With rapid improvement of the computing technologies, the expense of genome sequencing has been dropping [97]. Besides, more and more information technology (IT) has been applying to biomedicine, which makes patients achieve more health information. The process of personalized drug development requires the integration of medical data, as well as biological information, from diverse omics levels including DNA, RNA, proteins, networks, cells, metabolites, and tissues. More than just the integration of biomedical information, it also involves the regulation networks of multiple genes and targets. In other words, computational genomics, computational proteomics, computational regulation networks, and computational drug design play crucial and indispensable roles in the process of personalized medicine discovery. However, significant computation resources are required for the process. HPC infrastructure will play an increasingly important role in dealing with such tasks.

Big data in biomedicine, which refers to the increasing availability and explosive growth of medical data and biological information, provides an opportunity for future biomedical research and personalized medicine studies [97]. It also requires a new generation of HPC architectures and diverse technologies, data mining systems including omics information, inscription data, clinical data, and even financial data.

In conclusion, in the age of precision medicine and big data, opportunities and challenges exist together. HPC systems are increasingly essential components, which can compute, analyse, and integrate raw data efficiently, because of the large size of most biomedical data. In order to make personalized medicine more efficient and accurate, the tools and algorithms for integrating and analysing biomedical data need to be developed and improved [98].
